# Time Stand Still: Effects of Temporal Window Selection on Eye Tracking Analysis

**DOI:** 10.1525/collabra.25961

**Published:** 2021-07-29

**Authors:** Jonathan E. Peelle, Kristin J. Van Engen

**Affiliations:** 1 Department of Otolaryngology, Washington University in Saint Louis, MO, US,; 2 Department of Psychological and Brain Sciences, Washington University in Saint Louis, MO, US

**Keywords:** eyetracking, analytic flexibility, researcher degrees of freedom, gca, growth curve analysis, linear mixed effects analysis

## Abstract

The number of possible approaches to conducting and analyzing a research study—often referred to as researcher degrees of freedom—has been increasingly under scrutiny as a challenge to the reproducibility of experimental results. Here we focus on the specific instance of time window selection for time series data. As an example, we use data from a visual world eye tracking paradigm in which participants heard a word and were instructed to click on one of four pictures corresponding to the target (e.g., “Click on the hat”). We examined statistical models for a range of start times following the beginning of the carrier phrase, and for each start time a range of window lengths, resulting in 8281 unique time windows. For each time window we ran the same logistic linear mixed effects model, including effects of time, age, noise, and word frequency on an orthogonalized polynomial basis set. Comparing results across these time ranges shows substantial changes in both parameter estimates and p values, even within intuitively “reasonable” boundaries. In some cases varying the window selection in the range of 100–200 ms caused parameter estimates to change from positive to negative. Rather than rush to provide specific recommendations for time window selection (which differs across studies), we advocate for transparency regarding time window selection and awareness of the effects this choice may have on results. Preregistration and multiverse model exploration are two complementary strategies to help mitigate bias introduced by any particular time window choice.

## Introduction

“Often, the less there is to justify a traditional custom, the harder it is to get rid of it.”

- Mark Twain, *The Adventures of Tom Sawyer*

In recent years, researcher degrees of freedom—that is, the data collection and analysis choices made during the course of an experiment—have been increasingly in the limelight (Klein & Roodman, 2005; Simmons et al., 2011; Wicherts et al., 2016). A fair amount of focus has been on issues such as sample size, rules for stopping data collection, controlling for multiple statistical tests, and exclusion criteria for participants. Here we highlight an issue specifically related to the dependent measure for time series data: the temporal window over which data are analyzed.

There are many types of data involving large parameter spaces over which to search. In psychology and cognitive neuroscience, examples of time series signals include EEG, single unit recordings from neurons, and data from eye tracking and pupillometry. Functional MRI has faced this challenge both in the context of extended detection of three-dimensional signals (Worsley et al., 1992), and in the large number of potential analysis pipelines (Carp, 2012). Permutation testing has proven useful in detecting a variety of signals extended in both space (Nichols & Holmes, 2001; Smith & Nichols, 2009) and time (Maris & Oostenveld, 2007), but the challenges of large data sets with many possible analyses remain a contemporary issue. Thus, although in the current paper we focus on eye tracking data as an example, issues relating to data selection and model testing are broadly reflected in many areas of science.

**Figure 1.**
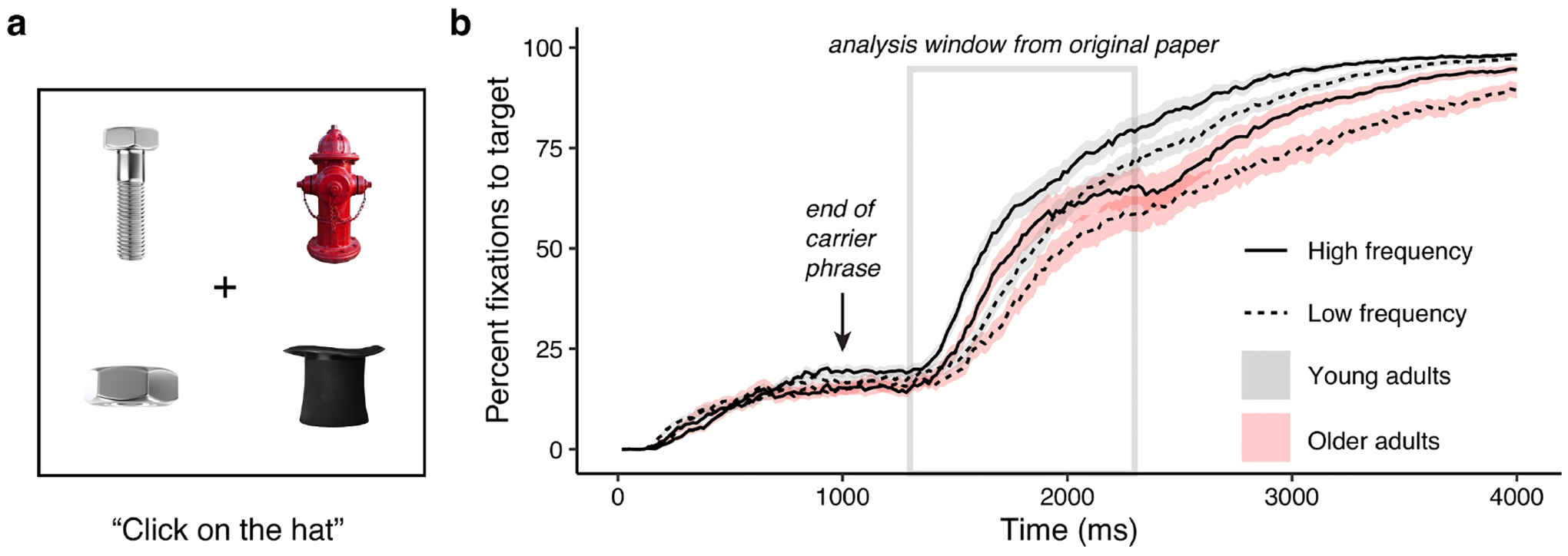
a) Illustrative display of a typical trial format from a visual world eye tracking study. The participant might hear “Click on the hat”, and the speed at which “hat” was looked at was taken as an index of spoken word recognition. b) Averaged data for young and older adults, for high and low frequency words, from Van Engen et al. (2020).

To investigate the effects of temporal window selection on time series analysis in eye tracking, we use data from a visual world paradigm experiment, which has a venerable history in psycholinguistic research (Allopenna et al., 1998; Cooper, 1974; Huettig et al., 2011). In a typical visual world paradigm experiment, multiple objects or words are shown on the screen (Figure 1a). A participant hears a word and moves a mouse to click on the target—but the gaze of the listener (rather than the mouse click) is used as an index of spoken word recognition. By averaging over trials in a condition, or across participants, a time course of target fixation—presumed to reflect lexical activation (Marslen-Wilson & Welsh, 1978; Treisman, 1960)—can be obtained (see Figure 1b).

A challenge for analyzing this type of eye tracking data is deciding the time window over which an analysis should be conducted. It takes approximately 200 ms to launch an eye movement (Hallett, 1986). Should the starting point for analysis be 200 ms after the word onset? After word offset? After a word’s perceptual center (Morton et al., 1976)? After the uniqueness point? Should the analysis continue for a set period of time, or until some proportion of the participants have looked to the target? For some duration after the word offset? For a particular shape of average response? Decisions made in individual papers are no doubt frequently well-reasoned. However, there is a lack of consensus over the “appropriate” time window, and similarly the degree to which the choice of a specific time window matters is unclear. Indeed, were we to ask for a show of hands among researchers indicating their reasoning for window selection, we suspect we would get a wide range of perspectives. Such variability leads to our main question: How much does the specific time window matter?

To answer this question, we take a data-driven approach to exploring the degree to which choices of time window can influence final results. We follow the spirit of sensitivity analyses (Saltelli et al., 2004) and multiverse analyses (Steegen et al., 2016) that look over a *set* of analyses (rather than a single analysis). The logic is that a raw data set does not give rise to a single analysis, but many possible analyses (and thus many possible results), depending on the choices made by researchers. Considering these various decisions can be thought of as a collection of many worlds, or a *multiverse* of statistical results. In the current paper we reanalyze data from Van Engen et al. (2020) using a large number of starting times and window durations (see also Sala-i-Martin, 1997). We use the same statistical model for each time window based on logistic linear mixed effects analyses (Barr, 2008; Mirman, 2014; Mirman et al., 2008) to see how time window selection affects results. Our goal is to provide a picture of the possible model results that could be obtained by the selection of different analysis windows.

Target fixation over the full data window we recorded—4 seconds—is shown in Figure 1b. In our previously-published analysis, we chose the time between 1300 ms and 2300 ms after the onset of the carrier phrase (because the carrier phrase was 1000 ms long, this window began 300 ms into the target word). We chose this window based on a combination of examining previous visual world paradigm papers and the shape of the data (i.e., avoiding flat sections at the beginning and end of the time window, which we thought would be poorly fit by our statistical models). However, the choice of whether to, say, analyze a 1000 ms long window or a 1100 ms long window felt rather arbitrary. Our anecdotal experience with this less-than-straightforward selection process motivated the current mission.

## Method

Data and analysis scripts are available from https://osf.io/7nhts/.

### Data

A full description of the method can be found in Van Engen et al. (2020). In brief, we conducted an experiment using the visual world paradigm to assess spoken word recognition. We used 200 words: 25 low-frequency targets, 25 high-frequency targets, and 150 middle-frequency distractors. All words referred to imageable nouns, depicted using a color picture on a white background. Each display occurred with the spoken instructions (carrier phrase) “Click on the ________”. Recordings were made by an American male from the Midwest. A single 1000 ms recording was used for the carrier phrase, and recordings of each target word were added. Half of the participants heard the stimuli in quiet, while the other half heard stimuli in steady speech-shaped noise at a signal-to-noise ratio (SNR) of +3 dB.

Participants were 41 young adults aged 18–25 years (25 female, M = 21.2, SD = 1.8) and 39 older adult counterparts aged 65–84 years (24 female, M = 71.7, SD = 5.1). We measured eye movements with a Tobii X120 eye tracker controlled by LabView 6.2 (RRID:SCR_014325) at a sampling rate of 60 Hz. Participants were seated approximately 0.5 meters from the camera eye. Informed consent was obtained under a protocol approved by the Washington University in Saint Louis Institutional Review Board.

### Statistical model

We used the same overall analysis framework as a prior study (Van Engen et al., 2020): logistic growth curve analysis to model the by-participant target fixation data using the *lme4* (Bates et al., 2015) and *lmerTest* (Kuznetsova et al., 2017) packages in R version 4.0.3 (RRID:SCR_001905) (R Core Team, 2020). We modelled the time course with a third-order orthogonal polynomial basis set (linear, quadratic, and cubic effects of time), along with fixed effects for age (young vs. older), frequency (high vs. low), and noise (quiet vs. noisy), and the interactions among these three factors. We also included participant and participant-by-frequency random effects in the model to capture individual differences and differences in the effect of the frequency manipulation on each participant. Statistical significance was determined using p values based on asymptotic Wald tests (the default in the *glmer* function from the *lme4* package in R). The model specification is as follows:


    m <- glmer(fixation ~ 
                    (linear+quadratic+cubic)*(age*noise *frequency) + 
                    (linear+quadratic | Subject) + 
                    (linear+quadratic+cubic | Subject:frequency),
                   data=df,
                   family=binomial, 
                   control=glmerControl(optimizer=“bobyqa”, optCtrl=list(maxfun=1e5))) 


Note that this model was chosen to match that in Van Engen et al. (2020), and thus in the current paper we did not explore variations in model specification.

### Window selection analysis

We examined a range of start times from 700–2200 ms (with target word onset occurring at 1000 ms), and for each start time a range of window lengths from 300–1800 ms, in steps of 16.667 ms (the sampling rate of the eye tracker). We purposefully chose these to span values larger than those typically used in the visual world paradigm literature in order to give a good sense of the parameter space (and to ensure that we captured the range of values typically used). This range also serves as a sanity check, in that we anticipated that including extreme values would lead to poor (or odd) model fits. We ran all combinations of the start times and window lengths for a total of 8281 models.

## Results

**Figure 2.**
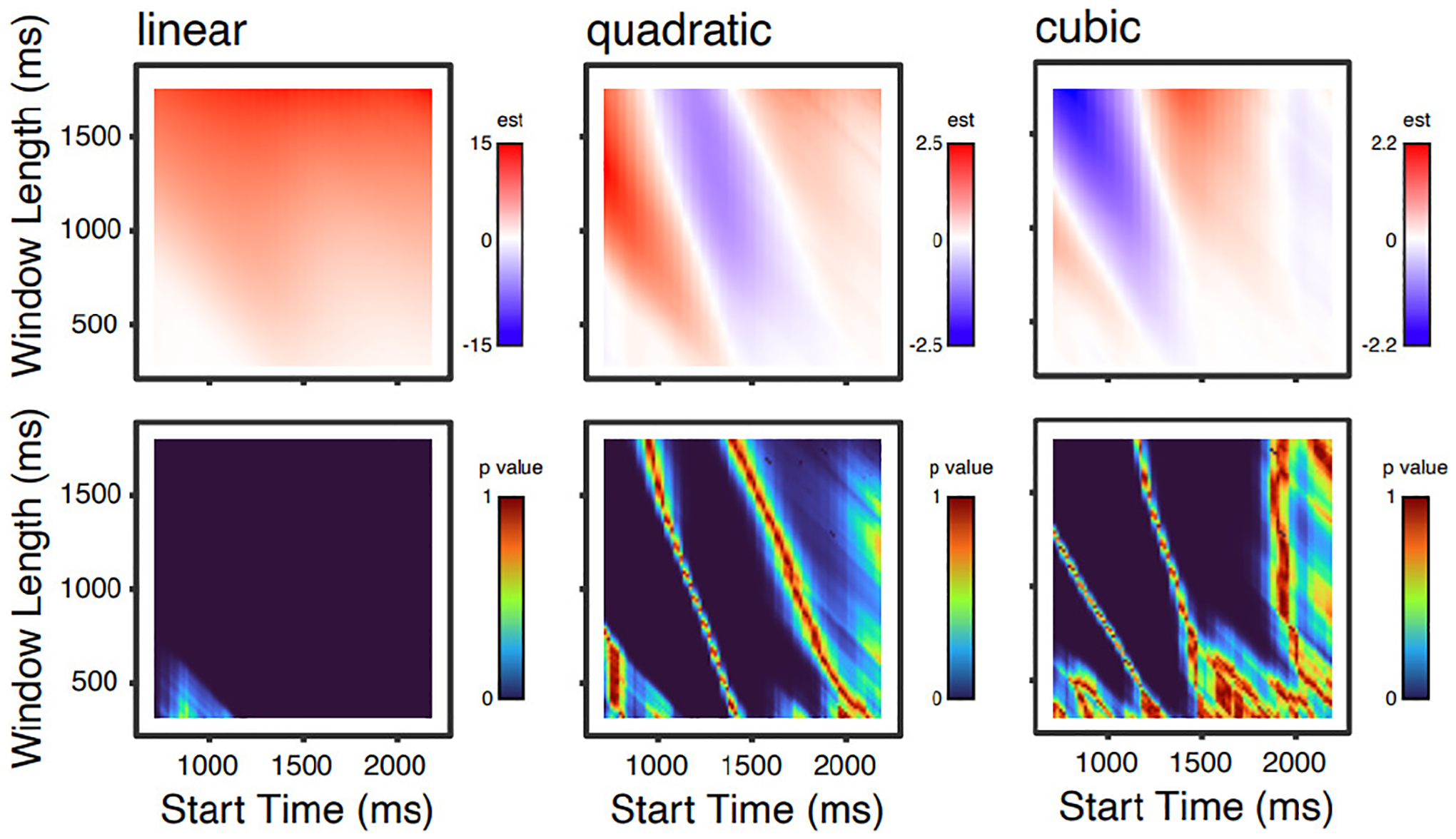
Parameter estimates (top) and p values (bottom) for 8281 models for linear, quadratic, and cubic effects of time.

**Figure 3.**
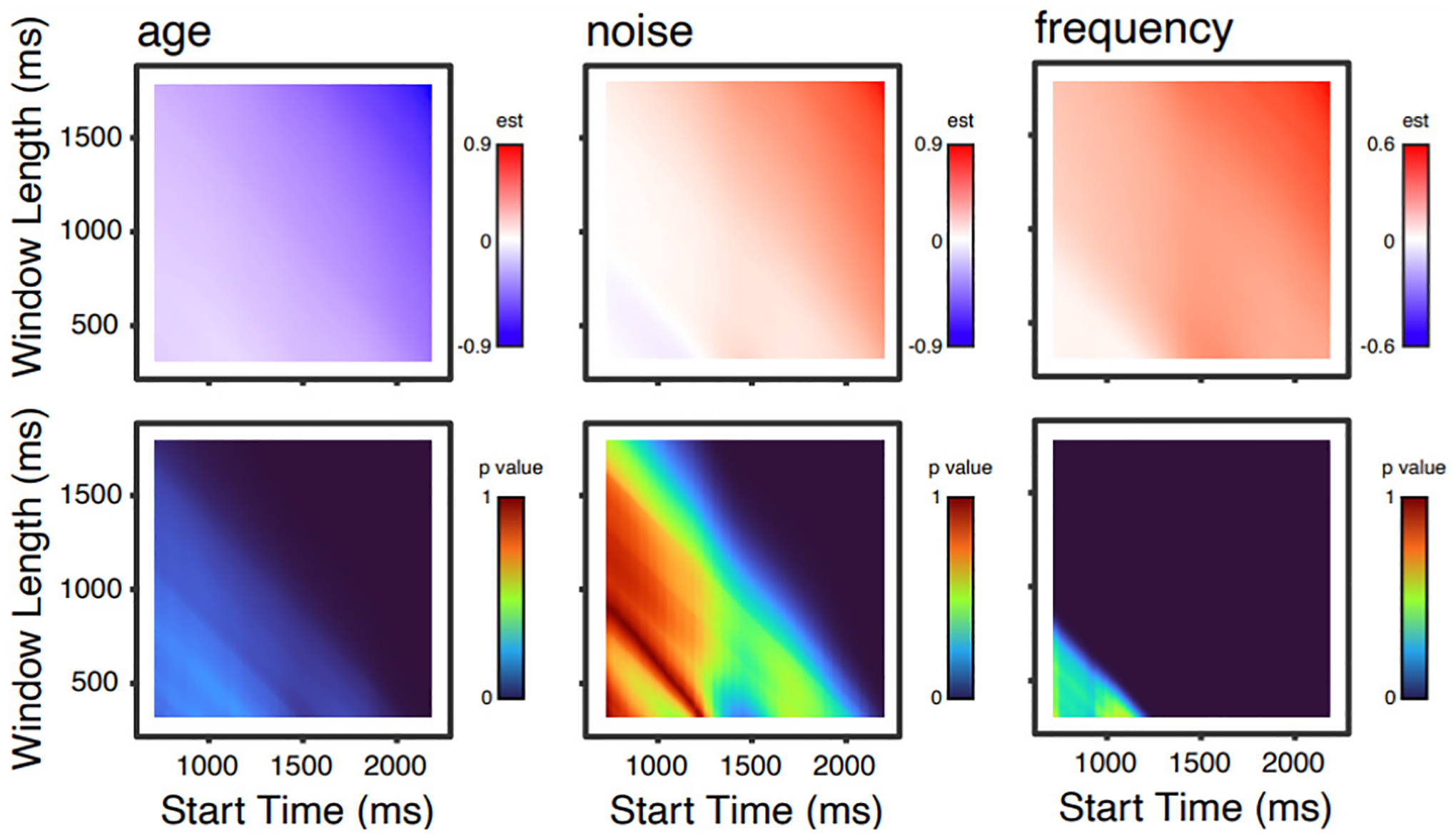
Parameter estimates (top) and p values (bottom) for 8281 models for main effects of age, noise, and word frequency.

**Figure 4.**
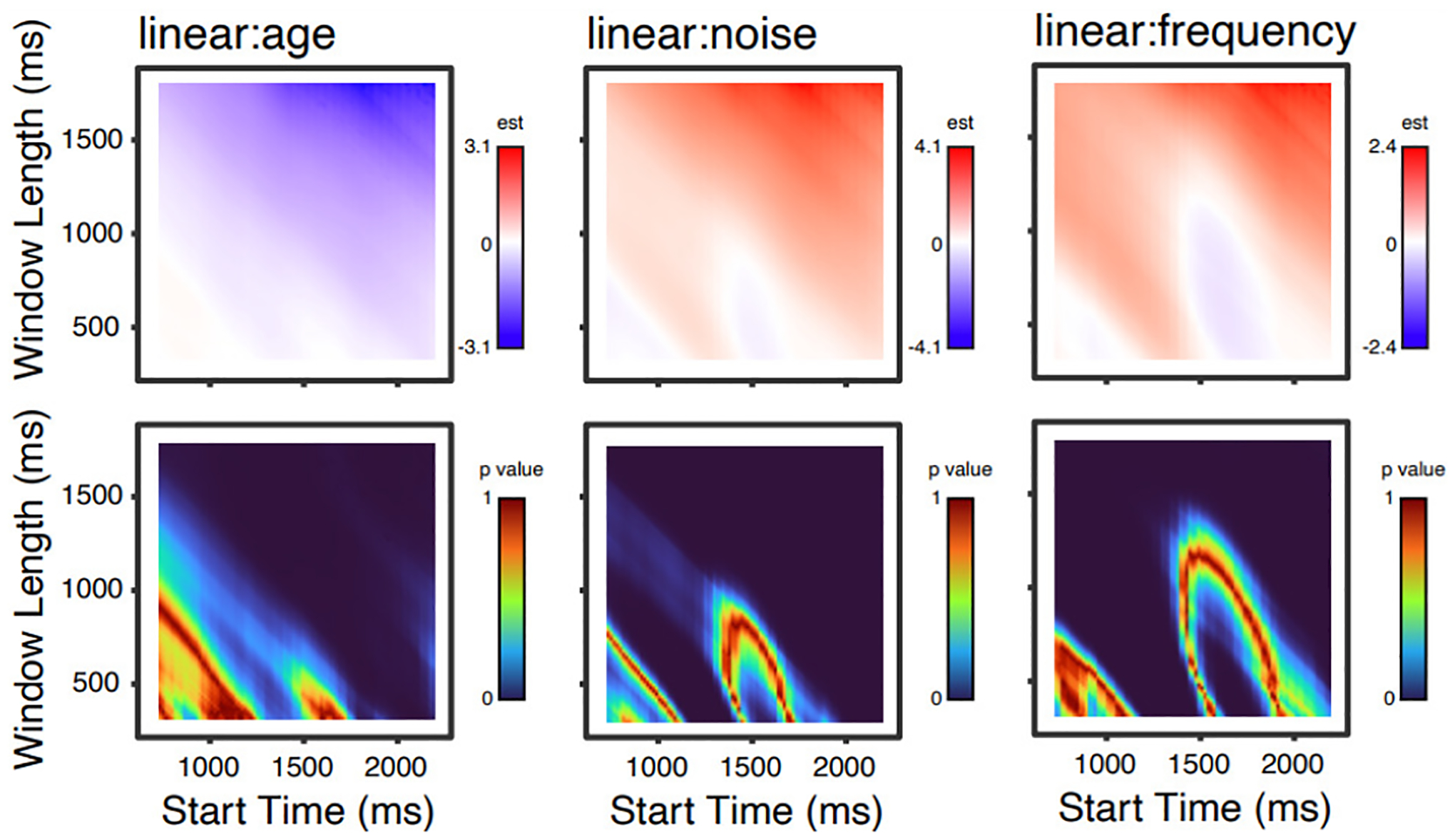
Parameter estimates (top) and p values (bottom) for 8281 models for linear effects of age, noise, and word frequency.

Figure 2 shows parameter estimates and p values for the three time parameters (orthogonalized linear, quadratic, and cubic effects of time); Figure 3 shows these values for main effects of age, noise and word frequency; and Figure 4 shows these values for linear effects of time and age, noise, and word frequency.

One pattern that is apparent in all of these analyses is that, although p values below 0.05 (i.e., “significant”, though see Cohen, 1994) are found over a wide range of time windows, several effects have ranges with high p values. For any given time window, one might interpret this lack of a significant effect differently knowing that shifting the time window by 100 ms would change the significance (compared to a situation in which varying the time window did not). Another point is that for many effects, given a fixed start time, increasing the window length increases sensitivity to effects (that is, longer time windows show p values that are consistently smaller than short time windows).

At first glance, it may also seem troubling that the magnitudes and even signs of parameter estimates are also changing as a function of time window. For example, the quadratic and cubic terms (Figure 2) both transition from positive to negative and back again (a red sector, a blue sector, another red sector). These changes correspond to inflection points in the curves: as the start time and/or window length are varied, the inflection points move around within the time window of the modeled data, such that over all models (as plotted here) “stripes” occur. Although considering the variability in parameter estimates is useful for illustrative purposes, in the context of GCA, a change in the sign or magnitude of a parameter estimate is not necessarily surprising or indicative of a problem. Rather, polynomial terms are simply adjusting to fit the overall shape, and inference is typically done by comparing models (deemphasizing the importance of a particular component of the model fit). It is also important to consider that polynomial basis functions will differ based on the number of time points of data being modeled (i.e., shorter time windows get assigned more extreme values).^1^ Thus, changes in magnitude of parameter estimates are not directly comparable across time windows of different lengths. Nevertheless, from a big-picture perspective, it is important to realize that different time window selections will influence model behavior, and, in some cases, study outcomes. Particularly, if authors have specific hypotheses about how fixed effects of interest (e.g., age) will interact with parameter estimates (e.g., linear), then time window selection will impact the magnitude and direction of these interactions.

**Figure 5.**
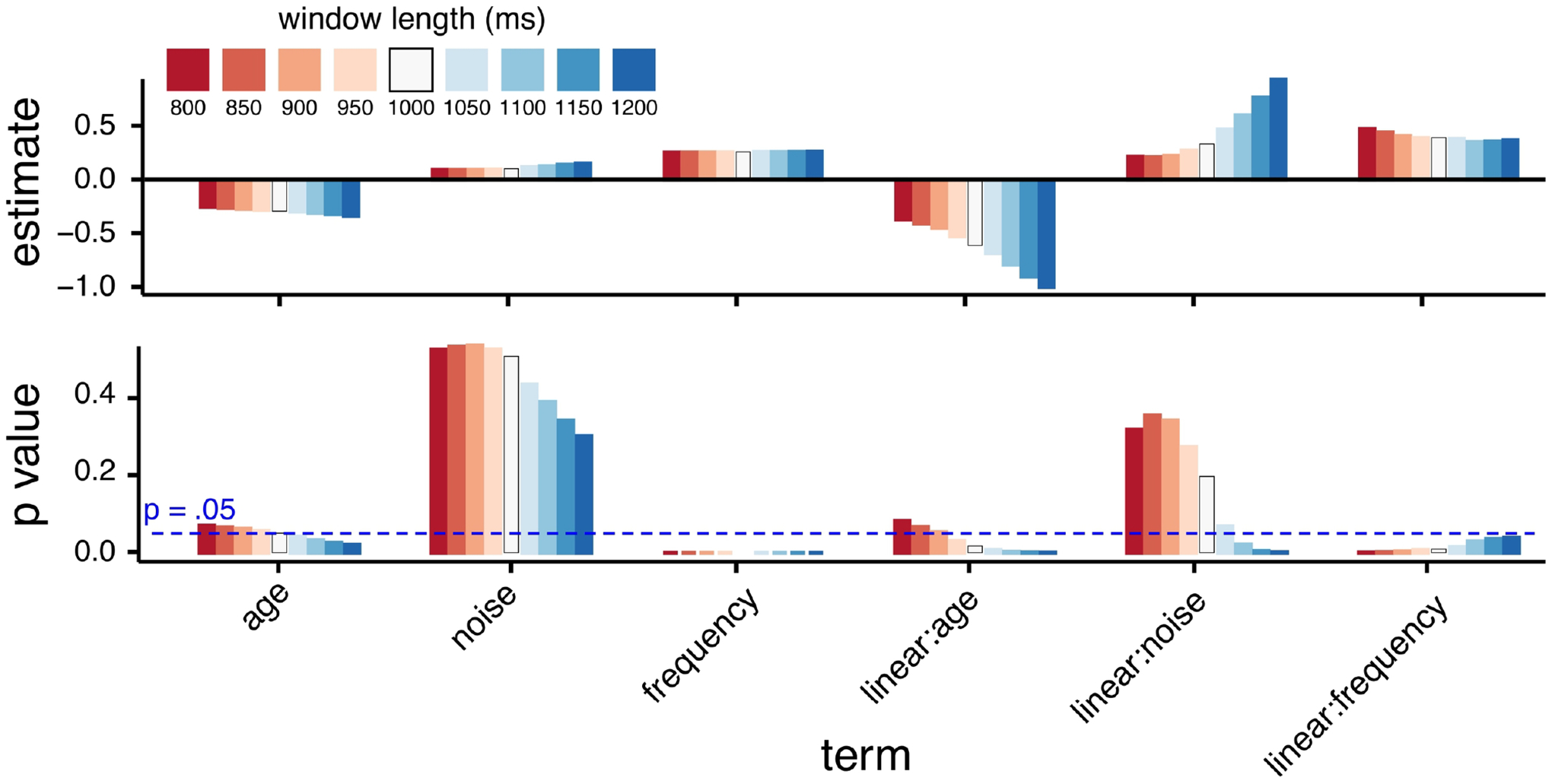
Illustration of how changing temporal window length affects results for a subset of models tested. In all examples, the starting time was 1300 ms (matching our published paper). In several cases, varying the window length between 800–1200 ms would result in different parameter estimates (top), and differing interpretations of statistical significance in a traditional null hypothesis testing framework (bottom), compared to the 1000 ms temporal window used in Van Engen et al. (2020).

As a final illustration, Figure 5 shows nine time windows that all share the starting time (1300 ms) of our published analysis, and window durations between 800–1200 ms, along with parameter estimates and p values (which are repeated from the more comprehensive plots in Figure 3 and Figure 4). Inspection of changes in p values in Figure 5 suggests that time window deviations as small as 50 ms, in a time range that might be used in an actual analysis, can lead to different model results. For example, the interaction of age and the linear effect of time is significant for a window length of 1000 ms, but not for 900 ms; the interaction of noise and the linear effect of time was *not* significant with a window length of 1000 ms, but would have been with a window length of 1100 ms; and so on.

## Discussion

For some experimental designs, the choice of what data to model is relatively straightforward. For time series analysis, however, the temporal window over which data are modeled is a key factor that is not always straightforward to choose. To explore the consequences of time window selection, we have used real data from a visual world paradigm eye tracking study to illustrate how changing the subdivisions of selected data can affect parameter estimates and statistical inference (here, p values). Our results show that changes in time window selection can indeed significantly affect the outcomes.

In the context of GCA-based analysis of our particular data set, we note that the significance of many effects increases with longer window lengths. Presumably, the orthogonalized polynomial time effects we used are better able to fit a longer time window, and these improved model fits are associated with lower overall p values. It may be that (at least in the context of visual world paradigm data) GCA analyses will generally benefit from slightly longer time windows, at least when orthogonalized polynomial effects of time are used to model the data (Mirman, 2014).

Of course, not all of the time windows we included are reasonable given what we know about the time course of spoken word recognition. That is, an informed researcher with experience in the field could narrow the potential time window of analysis to a number of options substantially smaller than 8281. However, perhaps closer to the heart of the matter, we note that models can change substantially over relatively small territories. For example, even when we used the same start time (1300 ms) as in the original analysis, changes to parameter estimates and p values occur—*presto!*—over durations between 800–1200 ms, which are reasonably similar to time windows used in other studies. Thus, *any* flexibility in time window selection has the potential for significantly affecting model fits and results.

It is worth emphasizing that in the specific context of GCA, we should probably not worry about individual contributions of higher-order polynomials, losing the forest for the trees. That is, it is the overall fit of the function that is of primary interest (the combination of different polynomial basis functions). Statistical evaluations are frequently performed by comparing models (deemphasizing the importance of any particular component of the model fit). We have included plots of individual parameter estimates to help illustrate how the models fit the data, but these should be interpreted in the context of the overall analytic framework being adopted.

Given the potential for analysis window selection to affect the outcome of eye tracking analyses, what can we as researchers do to manage these effects?

One clear recommendation is to decide on an analysis window before conducting the analysis, ideally documenting the chosen window through a public preregistration (Nosek et al., 2018). Although second nature to an increasing number of researchers, preregistration is a far cry from being universal. The advantage of preregistering a time window is it guards against adjusting an analysis to fit a desired outcome. Specifically, it might be tempting to adjust an analysis window within a “reasonable” range if a model doesn’t appear to be working, but this runs the danger of biasing an analysis towards a particular outcome (Kriegeskorte et al., 2009). A pre-chosen analysis window eliminates this source of bias. (Of course, other analysis windows can be tried, but a preregistration would make clear what window was chosen ahead of time and what exploratory windows were implemented later on.)

How should a time window for analysis be chosen? Past studies are helpful, but with different stimuli or different participants the specific time window is likely to differ. We offer three suggestions for how to deal with this. The first is to run a pilot study, independent of the main study, which can be used to explore the effect of window selection on the results and help researchers determine an appropriate analysis window ahead of time. Pilot data has the advantage of using (presumably) the same stimuli, design, equipment, and statistical approach as the main study. Lessons from this initial work can save many tears in the main experiment. Understandably, the extra time and resources involved mean that pilot studies are not always possible—not everyone has the big money to run multiple well-powered experiments. And, if not properly powered, parameter estimates, model fits, and p values from a pilot study may be unreliable. Nevertheless, pilot data may be useful in establishing whether a time window is reasonable, or to determine something like the average time at which an effect begins to occur (e.g., Barr, 2008).

A complementary approach to preregistration involves explorations of parameter space and design choices (time window space being one of many possible areas), either by researchers themselves or in collaboration with others. A number of fields have benefitted from a “many teams” analysis approach in which multiple research teams analyze the same dataset; convergence (or lack thereof) across analyses may indicate how reliable results are (Botvinik-Nezer et al., 2020; Silberzahn et al., 2018). It may also be that even a single research team running multiple models may provide additional information about the robustness of an effect to specific researcher choices (Sala-i-Martin, 1997; Steegen et al., 2016). Of course, statistical inference is limited due to the non-independence of the data and the large number of models run. However, there may be a place for such explorations to supplement main analyses (particularly if the main analyses were preregistered). To be clear, we are in no way advocating running a large number of models and reporting only a “favorable” outcome. Rather, we simply point to these types of multiverse analyses as one possible approach for understanding the effect of analysis choices on results. In these cases we advocate for a “show, don’t tell” approach: rather than simply describing a range of analyses, a visual representation of the results will go a long way towards helping readers understand what has been done.

Thirdly, it should also be considered whether using data-driven methods for selecting a time window of interest might be used, such as using a grand average across conditions to select a time window based on the shape of that average. These may be appropriate if noise does not systematically differ across conditions (Brooks et al., 2016, though see also Kriegeskorte et al., 2009).

Finally, it is worth noting there are other ways of approaching time series data that may reduce reliance on a specific analysis window. Seedorff and colleagues (2018), for example, suggest a bootstrapping approach, BDOTS, to estimate differences in time series data in a time-by-time basis, and would thus have different constraints than our function-based modeling approach. General additive models (van Rij et al., 2019) are able to fit a broader set of shapes than the polynomial-based GCA approach we use here, and as a result may be less sensitive to the specific shape of the data within a time window. Both approaches are probably less sensitive to specific time windows chosen and would likely work better with larger time windows. For example, in the GAM approach, researchers could choose the widest possible window and fit a smoothing spline, making inferences about when curves diverge with a difference smooth. In the BDOTS approach, bootstrapping can be used to determine when curves differ from each other. In fact, it may be possible to implement some aspects of these analyses in GCA using a difference estimate between conditions over time. Such an approach would reduce or remove the influence of time window selection on the process.

Although the best approach may differ across studies, being transparent about the time window selection—through preregistration, multiverse analysis, or simply discussing the thought process and windows tried—will go a long way towards increasing confidence in results from any analysis depending on selection of a time window, providing one little victory in the quest for robust and replicable analysis.

## Supplementary Material

1

## Data Availability

Data and analysis scripts are available from https://osf.io/7nhts/.

## Footnotes

1 We thank a reviewer for bringing this to our attention, as demonstrated in this snippet of R code, which shows that a smaller data frame has a range of −.39 to 0.39, and a longer one from −.038 to .038 (an order of magnitude of difference):

## References

[R1] Allopenna PD, Magnuson JS, & Tanenhaus MK (1998). Tracking the time course of spoken word recognition using eye movements: Evidence for continuous mapping models. Journal of Memory and Language, 38(4), 419–439. 10.1006/jmla.1997.2558

[R2] Barr DJ (2008). Analyzing “visual world” eyetracking data using multilevel logistic regression. Journal of Memory and Language, 59(4), 457–474. 10.1016/j.jml.2007.09.002

[R3] Bates D, Mächler M, Bolker BM, & Walker SC (2015). Fitting Linear Mixed-Effects Models Using lme4. Journal of Statistical Software, 67(1), 1–48. 10.18637/jss.v067.i01

[R4] Botvinik-Nezer R, Holzmeister F, Camerer CF, Dreber A, Huber J, Johannesson M, Kirchler M, Iwanir R, Mumford JA, Adcock RA, Avesani P, Baczkowski BM, Bajracharya A, Bakst L, Ball S, Barilari M, Bault N, Beaton D, Beitner J, … Schonberg T (2020). Variability in the analysis of a single neuroimaging dataset by many teams. Nature, 582(7810), 84–88. 10.1038/s41586-020-2314-9 32483374 PMC7771346

[R5] Brooks JL, Zoumpoulaki A, & Bowman H (2016). Data-driven region-of-interest selection without inflating Type I error rate. Psychophysiology, 54(1), 100–113. 10.1111/psyp.12682 28000250

[R6] Carp J (2012). On the plurality of (methodological) worlds: Estimating the analytic flexibility of fMRI experiments. Frontiers in Neuroscience, 6, 1–13. 10.3389/fnins.2012.00149 23087605 PMC3468892

[R7] Cohen J (1994). The earth is round (p<.05). American Psychologist, 49(12), 997–1003. 10.1037/0003-066x.49.12.997

[R8] Cooper RM (1974). The control of eye fixation by the meaning of spoken language: A new methodology for the real-time investigation of speech perception, memory, and language processing. Cognitive Psychology, 6(1), 84–107. 10.1016/0010-0285(74)90005-x

[R9] Hallett P (1986). Eye movements. In Boff KR, Kaufman L, & Thomas JP (Eds.), Handbook of Perception and Human Performance. Wiley. https://searchworks.stanford.edu/view/1602320

[R10] Huettig F, Rommers J, & Meyer AS (2011). Using the visual world paradigm to study language processing: A review and critical evaluation. Acta Psychologica, 137(2), 151–171. 10.1016/j.actpsy.2010.11.003 21288498

[R11] Klein JR, & Roodman A (2005). Blind analysis in nuclear and particle physics. Annual Review of Nuclear and Particle Science, 55(1), 141–163. 10.1146/annurev.nucl.55.090704.151521

[R12] Kriegeskorte N, Simmons WK, Bellgowan PSF, & Baker CI (2009). Circular analysis in systems neuroscience: The dangers of double dipping. Nature Neuroscience, 12(5), 535–540. 10.1038/nn.2303 19396166 PMC2841687

[R13] Kuznetsova A, Brockhoff PB, & Christensen RHB (2017). lmerTest Package: Tests in Linear Mixed Effects Models. Journal of Statistical Software, 82(13), 1–26. 10.18637/jss.v082.i13

[R14] Maris E, & Oostenveld R (2007). Nonparametric statistical testing of EEG- and MEG-data. Journal of Neuroscience Methods, 164(1), 177–190. 10.1016/j.jneumeth.2007.03.024 17517438

[R15] Marslen-Wilson WD, & Welsh A (1978). Processing interactions and lexical access during word recognition in continuous speech. Cognitive Psychology, 10(1), 29–63. 10.1016/0010-0285(78)90018-x

[R16] Mirman D (2014). Growth curve analysis and visualization using R. Chapman & Hall/CRC.

[R17] Mirman D, Dixon JA, & Magnuson JS (2008). Statistical and computational models of the visual world paradigm: Growth curves and individual differences. Journal of Memory and Language, 59(4), 475–494. 10.1016/j.jml.2007.11.006 19060958 PMC2593828

[R18] Morton J, Marcus S, & Frankish C (1976). Perceptual centers (P-centers). Psychological Review, 83(5), 405–408. 10.1037/0033-295x.83.5.405

[R19] Nichols TE, & Holmes AP (2001). Nonparametric permutation tests for functional neuroimaging: A primer with examples. Human Brain Mapping, 15(1), 1–25. 10.1002/hbm.1058 PMC6871862 11747097

[R20] Nosek BA, Ebersole CR, DeHaven AC, & Mellor DT (2018). The preregistration revolution. Proceedings of the National Academy of Sciences of the United States of America, 115(11), 2600–2606. 10.1073/pnas.1708274114 29531091 PMC5856500

[R21] R Core Team. (2020). R: A language and environment for statistical computing. http://r-project.org

[R22] Sala-i-Martin XX (1997). I Just Ran Four Million Regressions (No. 6252). National Bureau of Economic Research. 10.3386/w6252

[R23] Saltelli A, Tarantola S, Campolongo F, & Ratto M (2004). Sensitivity analysis in practice: A guide to assessing scientific models. John Wiley & Sons Ltd.

[R24] Seedorff M, Oleson J, & McMurray B (2018). Detecting when timeseries differ: Using the Bootstrapped Differences of Timeseries (BDOTS) to analyze Visual World Paradigm data (and more). Journal of Memory and Language, 102, 55–67. 10.1016/j.jml.2018.05.004 32863563 PMC7450631

[R25] Silberzahn R, Uhlmann EL, Martin DP, Anselmi P, Aust F, Awtrey E, Bahník Š, Bai F, Bannard C, Bonnier E, Carlsson R, Cheung F, Christensen G, Clay R, Craig MA, Dalla Rosa A, Dam L, Evans MH, Flores Cervantes I, … Nosek BA (2018). Many analysts, one data set: making transparent how variations in analytic choices affect results. Advances in Methods and Practices in Psychological Science, 1(3), 337–356. 10.1177/2515245917747646

[R26] Simmons JP, Nelson LD, & Simonsohn U (2011). False-positive psychology: Undisclosed flexibility in data collection and analysis allows presenting anything as significant. Psychological Science, 22(11), 1359–1366. 10.1177/0956797611417632 22006061

[R27] Smith SM, & Nichols TE (2009). Threshold-free cluster enhancement: Addressing problems of smoothing, threshold dependence and localisation in cluster inference. NeuroImage, 44(1), 83–98. 10.1016/j.neuroimage.2008.03.061 18501637

[R28] Steegen S, Tuerlinckx F, Gelman A, & Vanpaemel W (2016). Increasing transparency through a multiverse analysis. Perspectives on Psychological Science, 11(5), 702–712. 10.1177/1745691616658637 27694465

[R29] Treisman AM (1960). Contextual cues in selective listening. Quarterly Journal of Experimental Psychology, 12(4), 242–248. 10.1080/17470216008416732

[R30] Van Engen KJ, Dey A, Runge N, Spehar B, Sommers MS, & Peelle JE (2020). Effects of age, word frequency, and noise on the time course of spoken word recognition. Collabra: Psychology, 6(1), 17247. 10.1525/collabra.17247 34327298 PMC8318314

[R31] van Rij J, Hendriks P, van Rijn H, Baayen RH, & Wood SN (2019). Analyzing the Time Course of Pupillometric Data. Trends in Hearing, 23, 2331216519832483. 10.1177/2331216519832483 PMC6535748 31081486

[R32] Wicherts JM, Veldkamp CLS, Augusteijn HEM, Bakker M, van Aert RCM, & van Assen MALM (2016). Degrees of freedom in planning, running, analyzing, and reporting psychological studies: a checklist to avoid p-hacking. Frontiers in Psychology, 7, 1832. 10.3389/fpsyg.2016.01832 27933012 PMC5122713

[R33] Worsley KJ, Evans AC, Marrett S, & Neelin P (1992). A three-dimensional statistical analysis for CBF activation studies in human brain. Journal of Cerebral Blood Flow & Metabolism, 12(6), 900–918. 10.1038/jcbfm.1992.127 1400644

