# Supplementary material for: Time Stand Still: Effects of Temporal Window Selection on Eye Tracking Analysis
Source: Collabra Psychol. Author manuscript; Available in PMC 2022 Jan 11. (PMC8752058; doi:10.1525/collabra.25961)
Supplement: 1 [file NIHMS1746279-supplement-1.pdf]

SUPPLEMENTARY MATERIALS

Peer Review History

Download: https://collabra.scholasticahq.com/article/25961-time-stand-still-effects-of-temporal-window-selection-on-eye-tracking-analysis/attachment/65800.docx?auth_token=lkdfGEB06fXYgPqOpcet

Downloaded from http://online.ucpress.edu/collabra/article-pdf/7/1/25961/476786/collabra_2021_7_1_25961.pdf by guest on 29 July 2021
